# Elevations in Serum Dickkopf-1 and Disease Progression in Community-Dwelling Older Adults With Mild Cognitive Impairment and Mild-to-Moderate Alzheimer’s Disease

**DOI:** 10.3389/fnagi.2019.00278

**Published:** 2019-10-15

**Authors:** Laura Tay, Bernard Leung, Audrey Yeo, Mark Chan, Wee Shiong Lim

**Affiliations:** ^1^Department of General Medicine, Sengkang General Hospital, Singapore, Singapore; ^2^Department of Rheumatology, Allergy and Immunology, Tan Tock Seng Hospital, Singapore, Singapore; ^3^Health and Social Sciences, Singapore Institute of Technology, Singapore, Singapore; ^4^Institute of Geriatrics and Active Ageing, Tan Tock Seng Hospital, Singapore, Singapore; ^5^Department of Geriatric Medicine, Tan Tock Seng Hospital, Singapore, Singapore

**Keywords:** Dickkopf-1, mild cognitive impairment, Alzheimer’s disease, disease progression, inflammation

## Abstract

**Background:** Disruption of Wnt signaling has been implicated in dysfunctional synaptic plasticity, the degree of which correlates with Alzheimer’s disease severity. We sought to examine whether serum levels of Dickkopf-1 (Dkk-1), a Wnt antagonist, are associated with global disease progression in older adults with mild cognitive impairment (MCI) and mild-to-moderate AD.

**Methods:** We prospectively followed 88 older adults with MCI and mild-to-moderate AD attending a Memory Clinic. Cognitive performance, functional performance and neuropsychological symptoms were assessed at baseline and after 1 year. We reviewed neuroimaging for white matter changes and medial temporal atrophy, and performed ApoE genotyping at baseline. Serum Dkk-1 was assayed at baseline and 1 year, along with blood biomarkers of inflammation and endocrine dysfunction. We defined global disease progression (“progressors”) as an increase in Clinical Dementia Rating Sum-of-Boxes (CDR-SB) score by >2 points at 1 year.

**Results:** Fifteen (17.0%) participants had global disease progression. At baseline, there was no difference in cognitive performance and neuropsychiatric symptoms between groups, although progressors were more impaired in instrumental activities of daily living (*p* = 0.008). Progressors had significantly greater deterioration in cognitive performance (*p* = 0.002), with significantly worse functional performance and more severe neuropsychiatric symptoms (*p* = 0.042) at follow-up. Serum inflammatory and endocrine biomarkers at baseline and 1 year were similar between progressors and non-progressors. Serum Dkk-1 had increased significantly from baseline amongst progressors, while non-progressors exhibited decremental Dkk-1 over time (Dkk-1_change_: 354.304 ± 670.467 vs. −173.582 ± 535.676 ng/ml, *p* = 0.001). Adjusting for age, gender and baseline cognitive performance, incremental Dkk-1 independently predicted global cognitive decline (*p* = 0.012).

**Conclusion:** Our results suggest progressively dysfunctional Wnt signaling through Dkk-1 antagonism contributes to disease progression amongst older adults with MCI and mild-moderate AD.

## Introduction

Alzheimer’s disease (AD) is the most common form of dementia, with significant impact on the individual, caregiver, health systems and society. Current treatment strategies remain only symptomatic, with no known cure or disease-modifying therapy. While a natural trajectory of decline is anticipated, disease progression in AD is significantly heterogeneous with high variability in the rate of cognitive decline amongst afflicted persons (Lam et al., 2013). Risk factors for dementia have been widely reported in the literature, and a recent study suggested that rapid progression may be influenced by demographic and clinical factors as well as genetic interactions (Ferrari et al., 2018).

Synaptic dysfunction occurs early in the course of AD, before evidence of neuronal cell death, and the loss of synapses correlates best with cognitive decline (Terry et al., 1991; Palop and Mucke, 2010). Extracellular plaques of amyloid β (Aβ) and neurofibrillary tangles of hyperphosphorylated tau are the neuropathological hallmarks of AD and contribute to synaptic toxicity and neuronal loss. Wnt signaling has a key role in synaptic plasticity and memory, being neuroprotective against Aβ-induced toxicity, tau phosphorylation, neuroinflammation and apoptosis (Vallee and Lecarpentier, 2016; Tapia-Rojas and Inestrosa, 2018a). Dickkopf-1 protein (Dkk-1) is a secreted Wnt antagonist and found to be elevated in post-mortem brain samples from AD patients (Caricasole et al., 2004). Mouse models have demonstrated Dkk-1 as a critical participant in synaptic disassembly induced by Aβ, with short-term exposure to Aβ yielding increasing expression of Dkk-1 with consequent rapid synaptic loss (Purro et al., 2012). The observed enhanced working memory and memory consolidation in old mice deficient in Dkk-1 suggests that neutralization of Dkk-1 may be beneficial in counteracting age-related cognitive decline (Seib et al., 2013). There has been only one prior study that examined the relationship between circulating Dkk-1 levels and cognition in older adults, in which baseline Dkk-1 predicted decline in cognitive performance during follow-up (Ross et al., 2018). However, the study specifically recruited participants with subjective memory concerns and excluded those with confirmed clinical diagnoses of mild cognitive impairment (MCI) and dementia.

Dkk-1 appears to play a role in chronic inflammation (Diarra et al., 2007; Sato et al., 2010; Chae et al., 2016), which has been implicated in the neuropathological profile of AD (Uchihara et al., 1997; Vehmas et al., 2003). Plasma levels of inflammatory cytokines had also been associated with pathological severity, with significant elevations in peripheral inflammatory signals over time amongst AD patients who exhibited rapid cognitive decline (Leung et al., 2013).

The objective of this exploratory study was to investigate whether circulating Dkk-1 is associated with disease progression in older adults with MCI and mild-to-moderate Alzheimer’s dementia. In addition, we measured serum markers representing inflammation and the endocrine axes due to reported associations with AD. If established, the study findings would support the potential role of Dkk-1 antagonist molecules to ameliorate AD progression.

## Materials and Methods

### Study Population

This is a prospective study of community-dwelling older adults from a tertiary Memory Clinic in Singapore. We recruited 96 subjects with a diagnosis of MCI or mild-moderate Alzheimer’s dementia (AD) between December 2012 and November 2013. 88 participants completed a 1-year follow up.

Informed written consent was obtained from the patient or legally acceptable representative where appropriate. Ethics approval was obtained from the Domain Specific Review Board (DSRB) of the National Healthcare Group (NHG).

#### Diagnostic Categories

*MCI* was operationalized as follows: (1) global Clinical Dementia Rating (CDR) (Morris, 1993) score of 0.5; (2) presence of subjective memory complaint with corroboration by a reliable informant; (3) delayed recall >1 SD below the age and education-adjusted means of healthy community-dwelling subjects based on an earlier normative study (Sahadevan et al., 2002); (4) relatively normal general cognitive function, defined as Chinese Mini Mental State Examination (CMMSE) (Sahadevan et al., 2000) score ≥21 for subjects with ≤6 years education and ≥24 for those with >6 years of education; (5) largely intact activities of daily living; and (6) no clinical dementia.

*Mild-moderate AD* subjects were diagnosed according to National Institute of Neurological and Communicative Disorders and Stroke and the Alzheimer’s Disease and Related Disorders Association (NINCDS-ADRDA) criteria for *probable AD* (McKhann et al., 1984), with global CDR of 0.5, 1 or 2, for very mild, mild or moderate dementia, respectively. We excluded subjects with a diagnosis of *possible AD* in view of the confounding co-morbid diagnoses and differing clinical course in these individuals.

#### Eligibility Criteria

Potentially eligible subjects must have been aged >55 years, with a diagnosis of MCI or mild to moderate AD at baseline, community-dwelling, and accompanied by a reliable caregiver informant.

Subjects with presence of other central nervous conditions (stroke disease, Parkinson’s disease, subdural hematoma, normal pressure hydrocephalus, and brain tumor); presence of systemic conditions that can contribute to cognitive impairment (hypothyroidism, B12 deficiency, and hypercalcaemia); and presence of any active neuropsychiatric conditions producing disability were excluded. Residents of sheltered or nursing homes were also excluded.

The validity of the overall cognitive evaluation process and CDR scoring has been previously established (Chong and Sahadevan, 2003; Lim et al., 2005). All patients had undergone laboratory investigations and neuroimaging to exclude potentially reversible causes of dementia. All cases were discussed in a multidisciplinary consensus meeting in which all relevant results were reviewed for accurate clinical phenotyping. Patients meeting study eligibility criteria were then recruited.

### Measures

#### Cognitive Assessment

We used the CDR (Morris, 1993), a structured clinician rating, to assess cognitive change and determine dementia severity. The CDR is a global dementia rating scale encompassing assessment across six domains – memory, orientation, judgment-problem solving, community affairs, home hobbies, and personal care – providing both a global and sum-of-boxes (CDR-SB) score (range 0 to 18). Global CDR 0 indicates no dementia, and CDR 1, 2 and 3 correspond to mild, moderate, and severe dementia, respectively. A global CDR stage of 0.5 can be representative of either very mild dementia or MCI, with the latter diagnosis being assigned when the cognitive impairment does not fulfill dementia criteria. CDR-SB provides a finer gradation of impairment and has demonstrated sensitivity to progression in dementia (Williams et al., 2013). The attending geriatrician, trained in administration of the CDR, rated each patient’s CDR at baseline and 1-year follow-up. We defined disease progression as an increase ≥2 points from baseline on the CDR-SB, based on an observed annual rate of change in CDR-SB score of 1.91 ± 0.07 amongst participants with mild AD (Williams et al., 2013).

Cognitive performance was assessed using the Chinese Mini-Mental State Examination (CMMSE). MCI subjects also underwent a neuropsychological assessment, which was modeled after the Consortium to Establish a Registry for Alzheimer’s Disease (CERAD) psychometric instrument, with local validation and education adjustment (Sahadevan et al., 2002). The battery of tests provides for assessment across multiple cognitive domains – memory (word list for immediate and delayed recall, and recognition memory), language (category fluency and modified Boston Naming Test), executive function (category fluency and Color Trail 2), and visuo-spatial ability (Block Design subtest of the Weschler Adult Intelligence Scale-revised).

#### Blood Biomarkers

All subjects underwent blood biomarker measurements at baseline and 1 year. Participants fasted for 8 h before the blood draw, and the aliquoted serum was frozen and stored at −80°C until the tests were performed. Inflammatory status was assessed by serum levels of Dkk-1, soluble tumor necrosis factor-α receptor-1 (TNF-R1, both R&D Systems, Minneapolis, MN, United States), and interleukin-6 (high sensitive IL-6, eBioscience, San Diego, CA, United States) via ELISA. The endocrine markers insulin-like growth factor-1 (IGF-1) and dehydroepiandrosterone sulfate (DHEA-S) were quantified using commercial ELISA assays (BioVendor, Brno, Czech Republic and Abcam, Cambridge, United Kingdom, respectively). All biomarkers were measured in duplicates according to manufacturers’ recommendations, and the average value was reported for all assays. Detection limits were as follows: DHEAS, 0.2 μmol/L; Dkk-1, 30 pg/ml; IGF-1, 2 ng/ml; IL-6, 0.1 pg/ml; TNF-R1, 30 pg/ml.

#### Other Co-variates

Demographic data and co-morbid vascular risk factors - hypertension, hyperlipidemia, diabetes mellitus, atrial fibrillation, peripheral vascular disease, smoking history, and ischemic heart disease – were documented at baseline. We reviewed participants’ medical records for the presence of chronic inflammatory disease and any active treatment with steroids or immunosuppressant medication.

At both baseline and 1-year follow-up, functional performance was evaluated using Barthel’s basic activities of daily living (ADL) index (Mahoney and Barthel, 1965) and Lawton and Brody’s instrumental ADL (iADL) index (Barberger-Gateau et al., 1992), while severity of neuropsychological symptoms was assessed using the Neuropsychiatric Inventory Questionnaire (Cummings, 1997).

We reviewed each subject’s neuroimaging scan - brain computed tomography (CT) scan (27 subjects) or magnetic resonance imaging (MRI) (68 subjects). White matter lesion (WML) severity was graded using the Age-Related White Matter Changes (ARWMC) scale applicable to both CT and MRI (using T2-weighted axial slices), in five different regions and separately for the right and left hemispheres – frontal area, parieto-occipital area, temporal area, infra-tentorial area, and basal ganglia. Each region was graded on a 4-point scale, and the global white matter score derived from summation of the individual scores (range 0–30), with higher ARWMC score reflecting a greater burden of white matter lesions (Wahlund et al., 2001). Medial temporal atrophy (MTA) score reflecting neurodegeneration was scored on T1-weighted coronal slices for MRI or non-enhanced CT, parallel to the brainstem axis and perpendicular to the hippocampal axis, by a consensus method where the scores range from 0 (no atrophy) to 4 (severe atrophy) (Wahlund et al., 2000). Visual ratings for ARWMC and MTA were performed by a blinded rater.

APOE genotyping into *APOE*ε*2,3,4* isoforms was performed via restriction enzyme analysis using the Applied Biosystems platform ABI Prism 310 Genetic Analyzer.

### Statistical Analyses

Descriptive data are presented as means (± SD) or median (interquartile range, IQR) for quantitative variables and as absolute and relative frequencies for categorical variables. We performed univariate analyses comparing progressors and non-progressors in baseline demographics, co-morbidities and neuroimaging markers, along with changes in cognitive, functional performance and blood biomarker measures, using independent-sample *t*-test and Wilcoxon Rank-Sum test for parametric and non-parametric continuous variables, respectively, and Chi-square test for categorical variables. Pearson’s correlation was performed to examine the relationship between changes in individual blood biomarkers and cognition as measured on both CMMSE and CDR-SB, for the overall cohort as well as subgroup analyses by baseline diagnosis (MCI or AD).

Multiple logistic regression, with disease progression (CDR-SB increase >2 points) as the outcome variable, was performed to examine the independent role of blood biomarkers. The full model included *a priori* age, gender, and baseline cognitive performance, in addition to each predictor biomarker showing significant univariate association with the outcome of interest. The chosen variables were guided by our sample size and prior literature on factors potentially influencing disease progression in AD, while ensuring no multi-collinearity.

Statistical analyses were performed using SPSS version 24. All statistical tests were two-tailed, with *p-*value <0.05 considered statistically significant. Owing to the number of blood biomarkers examined in our correlation analyses, we applied Bonferroni correction (0.05 divided by 5 blood biomarkers) to control for family wise error rate, such that only a *p*-value <0.01 would be considered significant for blood biomarker changes.

## Results

### Clinical Characteristics (Table 1)

The mean age of our cohort was 77.0 ± 6.8 years, with 15 (15.6%) having a diagnosis of MCI, 69 (71.9%) mild AD, and 12 (12.5%) moderate AD. Eighty-eight (91.7%) of the 96 participants completed 1-year follow-up, of whom 14 had MCI and 74 had mild-moderate AD at baseline. There was no significant difference in age, gender, and baseline cognitive performance between participants who completed vs. those who were lost to follow-up.

**TABLE 1:** Clinical characteristics and disease progression.

	**Progressors *N* = 15**	**Non-progressors *N* = 73**	***p*-value**
**Demographics**			
Age	76.2 (6.2)	76.9 (6.7)	0.726
Gender (Female)	11 (73.3%)	47 (64.4%)	0.505
Ethnicity (Chinese)	14 (93.3%)	68 (93.2%)	0.745
Education (years)	5.8 (5.1)	5.9 (4.5)	0.949
**Comorbidities**			
Hypertension	11 (73.3%)	47 (64.4%)	0.505
Diabetes mellitus	3 (20%)	24 (32.9%)	0.325
Hyperlipidemia	7 (46.7%)	47 (64.4%)	0.199
Ischemic heart disease	1 (6.7%)	14 (19.2%)	0.451
Atrial fibrillation	0	2 (2.7%)	1.00
Stroke/TIA	0	3 (4.1%)	1.00
**Baseline diagnosis**			0.540
MCI	1 (6.7%)	13 (17.8%)	
Mild AD	12 (80%)	53 (72.6%)	
Moderate AD	2 (13.3%)	7 (9.6%)	
**Cognitive performance**			
CMMSE_baseline_	16.5 (4.7)	18.7 (4.9)	0.116
CMMSE_1–yr_	13.0 (6.4)	18.2 (5.7)	0.003^∗∗^
CMMSE_change_	−3.6 (3.2)	−0.6 (3.1)	0.002^∗∗^
**Mood**			
Depression_baseline_	0	2 (2.7%)	1.00
Depression_1–yr_	0	0	
**Behavioral**			
NPI-sev_baseline_	5.3 (5.1)	3.4 (3.3)	0.198
NPI-sev_1–yr_	7.0 (6.8)	3.0 (4.0)	0.042^∗^
NPI-sev_change_	1.7 (4.7)	−0.4 (3.2)	0.033^∗^
**Functional**			
MBI_baseline_	100 (90–100)	100 (95–100)	0.092
MBI_1–yr_	95 (90–100)	100 (95–100)	0.019^∗^
MBI_change_	0 (−5–0)	0 (−2.5–0)	0.234
iADL_baseline_	12.5 (3.4)	15.6 (5.4)	0.008^∗∗^
iADL_1–yr_	9.1 (4.3)	14.5 (5.5)	0.001^∗∗^
iADL_change_	−*3*.*4*(*4*.*3*)	−*8*.*2* (*1*.*1*)	0.059
**Neuroimaging**			
ARWMC total (*n* = 86)	5.31 (5.98)	5.71 (4.03)	0.405
MTA (*n* = 83)	1.46 (1.13)	1.51 (1.03)	0.868
ApoE4 positive	6 (40%)	25 (34.7%)	0.654
Use of cognitive enhancer	13 (86.7%)	55 (76.4%)	0.506

*AD, Alzheimer’s disease; ARWMC, age-related white matter changes; CMMSE, Chinese Mini Mental State Examination; iADL, Lawton and Brody’s instrumental activities of daily living; MBI, Modified Barthel Index; MCI, mild cognitive impairment; MTA, medial temporal atrophy; NPI, neuropsychiatric inventory questionnaire. Mean (SD) or median (IQR), unless otherwise indicated. ^∗^*P* < 0.05; ^∗∗^*P* < 0.01.*

Fifteen (17.0%) of 88 participants who completed 1-year follow-up exhibited global disease progression. Fourteen (18.9%) of participants with mild-moderate AD at diagnosis fulfilled CDR-SB cut-off for progression, while only 1 participant (7.1%) in the MCI group had progressed.

Baseline cognitive diagnosis (MCI, mild or moderate AD), cognitive performance and severity of neuropsychiatric symptoms were similar between participants with and without disease progression. However, progressors had significantly greater functional impairment in instrumental ADLs at baseline (15.6 ± 5.4 vs. 12.5 ± 3.4, *p* = 0.008) and follow-up. At 1-year follow-up, there was significantly greater decline in cognitive performance and increased severity of neuropsychiatric symptoms amongst progressors compared with non-progressors.

Vascular burden, as reflected by comorbidities and extent of white matter lesions on neuroimaging, was similar between progressors and non-progressors. There was no difference in severity of hippocampal atrophy at baseline, and ApoE-4 status was similar, between progressors and non-progressors.

### Blood Biomarkers

#### Association of Disease Progression With Blood Biomarkers at Baseline and 1 Year (Table 2)

While there was no significant difference in baseline Dkk-1, serum Dkk-1 at 1 year was significantly higher in progressors compared with non-progressors (1045.762 ± 553.839 vs. 714.429 ± 366.852, *p* = 0.005). Contrary to the observed decremental Dkk-1 at 1 year relative to baseline amongst non-progressors, Dkk-1 had increased significantly from baseline amongst progressors (DKK-1_change_: 354.304 ± 670.467 vs. -173.582 ± 535.676, *p* = 0.001).

**TABLE 2:** Blood biomarkers and disease progression.

	**Progressors *N* = 15**	**Non-progressors *N* = 73**	***p*-value**
IL6_baseline_ (pg/ml)	0.170(0.060−0.262)	0.227(0.105−0.506)	0.190
IL6_1–yr_ (pg/ml)	0.420(0.290−0.668)	0.500(0.300−1.582)	0.424
IL6_change_ (pg/ml)	0.308(0.140−0.450)	0.251(0.058−0.564)	0.786
TNFR1_baseline_ (pg/ml)	6858.91(4791.03−9214.85)	6929.04(5248.25−8648.16)	0.837
TNFR1_1–yr_ (pg/ml)	2497.58(1713.54−3483.35)	3465.24(1978.96−4638.76)	0.146
TNFR1_change_ (pg/ml)	−4689.65(−7501.31−490.83)	−4429.02(−6298.33−329.34)	0.606
IGF1_baseline_ (ng/ml)	129.25(72.43−136.84)	110.74(72.38−167.58)	0.960
IGF1_1 year_ (ng/ml)	87.118(75.787−134.751)	102.235(75.091−152.205)	0.706
IGF1_change_ (ng/ml)	−12.33(−53.745−5.504)	−3.281(−27.381−18.614)	0.434
DHEAS_baseline_ μmol/L	1.387(0.700−2.193)	1.865(1.056−2.979)	0.310
DHEAS_1–yr_ μmol/L	1.552(0.773−2.122)	1.943(1.176−3.100)	0.136
DHEAS_change_ μmol/L	0.025(−0.830−0.502)	0.187(−0.621−0.721)	0.534
Dkk-1_baseline_ (pg/ml)	611.270(493.09−855.650)	824.790(687.335−1057.795)	0.064
Dkk-1_1–yr_ (pg/ml)	1021.120(863.560−1317.650)	731.180(419.931−1047.955)	0.024^∗^
Dkk-1_change_ (pg/ml)	487.610(24.046−899.060)	−142.910(−605.305−179.605)	0.003^∗∗^

*DHEAS, dehydroepiandrosterone sulfate; Dkk-1, Dickkopf-1; IGF1, insulin-like growth factor-1; IL6, interleukin-6; TNFR1, tumor necrosis factor-α receptor-1. ^∗^*P* < 0.05; ^∗∗^*P* < 0.01.*

Other serum inflammatory and endocrine biomarkers – both at baseline and 1 year – were similar between progressors and non-progressors. There was also no difference in magnitude of individual inflammatory (IL-6 and TNF-R1) and endocrine biomarker change from baseline to 1 year between progressors and non-progressors.

#### Correlation Between Changes in Blood Biomarkers and Cognitive Performance (Table 3)

In analysis for the overall cohort of 88 participants, we observed no significant correlation between decline in CMMSE score and magnitude of change in individual biomarkers over time. Amongst the individual blood biomarkers, only incremental serum Dkk-1 over time correlated significantly with change in CDR sum-of-boxes score (*r* = 0.275, *p* = 0.010).

**TABLE 3:** Correlation between changes in blood biomarkers and cognitive performance.

	**Overall cohort (*N* = 88)**		**MCI (*N* = 14)**		**AD (*N* = 74)**	
	**CMMSE change (*r*, *p* value)**	**CDR-SB change (*r*, *p* value)**	**CMMSE change (*r*, *p* value)**	**CDR-SB change (*r*, *p* value)**	**CMMSE change (*r*, *p* value)**	**CDR-SB change (*r*, *p* value)**
IL6_change_	−0.130 (0.233)	0.002 (0.983)	0.093 (0.751)	0.110 (0.709)	−0.232 (0.050)	0.023 (0.899)
TNFR1_change_	−0.093 (0.395)	−0.012 (0.915)	−0.199 (0.494)	0.284 (0.325)	−0.077 (0.521)	−0.054 (0.647)
IGF-1_change_	−0.017 (0.879)	−0.055 (0.609)	−0.056 (0.849)	0.141 (0.630)	−0.018 (0.884)	−0.068 (0.567)
DHEAS_change_	0.102 (0.352)	−0.072 (0.506)	−0.059 (0.841)	−0.300 (0.298)	0.089 (0.457)	−0.032 (0.785)
Dkk-1_change_	−0.137 (0.208)	0.275 (0.010)^∗^	−0.357 (0.210)	−0.125 (0.295)	−0.125 (0.295)	0.346 (0.003)^∗∗^

*DHEAS, dehydroepiandrosterone sulfate; Dkk-1, Dickkopf-1; IGF1, insulin-like growth factor-1; IL6, interleukin-6; TNFR1, tumor necrosis factor-α receptor-1. ^∗^*P* < 0.05; ^∗∗^*p* < 0.01.*

In subgroup analysis amongst MCI participants, there was no correlation between any blood biomarker with either CMMSE or CDR-SB score change. However, we observed significant moderate correlation between incremental serum Dkk-1 and progressively higher CDR-SB scores over time in participants with mild-moderate AD at diagnosis (*r* = 0.346, *p* = 0.003).

#### Multiple Logistic Regression Model for Global Cognitive Decline (Table 4)

In multiple logistic regression, adjusting for age, gender, and baseline cognitive performance, incremental Dkk-1 over time independently predicted global disease progression. Incremental Dkk-1 within the upper quartile conferred 4.91-fold higher odds (95% confidence interval: 1.43–16.93, *p* = 0.012) for global cognitive decline.

**TABLE 4:** Multiple logistic regression for global cognitive decline.

	**Overall cohort (*N* = 88)**		**AD (*N* = 74)**	
	**OR (95% CI)**	***p*-value**	**OR (95% CI)**	***p*-value**
Age	1.002 (0.917–1.093)	0.972	0.968 (0.883–1.062)	0.489
Female	1.015 (0.258–3.989)	0.983	1.448 (0.323–6.499)	0.629
Baseline CMMSE	0.906 (0.793–1.036)	0.149	0.929 (0.801–1.077)	0.527
Dkk-1_change (top quartile)_	4.913 (1.426–16.929)	0.012^∗^	3.960 (1.074–14.593)	0.039^∗^

*Overall cohort: *R*^2^ = 0.169; AD: *R*^2^ = 0.142. ^∗^*P* < 0.05.*

We repeated the multiple logistic regression model for the subgroup with diagnosis of mild-moderate AD at baseline. Incremental Dkk-1 in the upper quartile significantly increased the odds for disease progression (Odds ratio = 3.96, 95% confidence interval: 1.07–14.39, *p* = 0.039). As only 1 MCI participant exhibited CDR-SB cut-off for progression, subgroup analysis was not performed for MCI.

## Discussion

This is the first study to demonstrate an association between circulating Dkk-1 and progressive decline in a cohort of older adults with MCI and mild-moderate AD. Deterioration in cognitive performance was paralleled by greater dependence in functional performance and increased neuropsychological symptoms, supporting the role of Dkk-1 in overall disease progression, particularly with established Alzheimer’s dementia. Notably, there was no difference in baseline vascular burden, hippocampal atrophy, and ApoE-4 status, nor was there a difference in other inflammatory and endocrine blood biomarkers.

The Wnt signaling pathway is fundamental to the development of the central nervous system and also plays critical roles in the adult brain, having been implicated in synaptic plasticity and cognition (Ortiz-Matamoros et al., 2013; Inestrosa and Varela-Nallar, 2014). Inhibition of Wnt signaling in mouse models of AD has been associated with accelerated onset and progression of AD neuropathology and memory loss (Tapia-Rojas and Inestrosa, 2018b). As a negative modulator of Wnt signaling, Dkk-1 has been demonstrated to trigger synaptic loss mediated by amyloid-β (Purro et al., 2012). Mouse models suggest that Dkk-1 increases with age and its deletion attenuates age-related cognitive decline (Seib et al., 2013). To date, there is only one other study which examined serological Dkk-1 in a cohort of older adults with subjective cognitive concerns but in the absence of clinically diagnosed dementia (Ross et al., 2018). Although baseline Dkk-1 was negatively related to the annual rate of change in global cognition, this association between Dkk-1 and cognition was however lost following exclusion of participants who developed dementia during follow-up, suggesting that Dkk-1 may be more predictive of dementia than mere cognitive change. Our results complement this earlier study by examining a cohort of older adults across the spectrum of cognitive impairment ranging from MCI to mild-moderate AD. Increased Dkk-1 over time correlated with progressive disease severity as indicated by the change in CDR-SB scores. Specifically, our subgroup analysis also showed incremental Dkk-1 was significantly associated with progressively higher CDR-SB scores amongst participants with mild-moderate dementia, but not at the MCI stage. The apparent lack of correlation between changes in Dkk-1 and disease progression in MCI may be attributable to the slower natural progression in MCI compared with dementia given the relatively short duration of follow-up in our study (Storandt et al., 2002), as well as the etiological heterogeneity of MCI, although patients with non-amnestic presentations as well as central nervous or systemic conditions that could potentially contribute to cognitive impairment were excluded. In line with the present findings, Dkk-3 - a related Dkk-1 protein - was also noted to be elevated in both plasma and cerebrospinal fluid of patients with AD (Zenzaimer et al., 2009), although its action on the Wnt-pathway is context-dependent and its induction of vascular endothelial growth factor with dual effects in the central nervous system may occur independently of the Wnt signaling pathway (Busceti et al., 2018*).* Interestingly, we observed decremental Dkk-1 over time amongst non-progressors, which could not be accounted for by cognitive enhancer use or clinical comorbidities, although our data limits further exploration for possible mechanisms for restoration of Wnt-activity. With synaptic failure underlying the cognitive manifestations of AD, our data suggests that Dkk-1 may contribute to AD progression through synaptic dysfunction consequent to Wnt antagonism.

Contrary to the established role of inflammation in pathogenesis of AD (Perry et al., 2001; Heppner et al., 2015; Bagyinszky et al., 2017), we found no association between blood biomarkers of inflammation and global cognitive decline in our cohort of cognitively impaired older adults. Despite elevated concentrations of inflammatory markers within senile plaques and neurofibrillary tangles (Hosfield and Humpel, 2015), studies examining circulating levels of inflammatory markers and cognitive decline or clinical AD have had conflicting results (Tan et al., 2007; Sundelof et al., 2009). Furthermore, clinical trials of non-steroidal anti-inflammatory drugs have failed to demonstrate benefit on both risk and progression of AD (Aisen et al., 2003; Szekely et al., 2008). In the current study, the lack of relationship between peripheral inflammatory markers and disease progression may be attributed to the effect of the blood-brain barrier such that circulating inflammatory biomarkers may have limited utility in reflecting the changes occurring within the brain.

Disease progression in MCI and mild-moderate AD was also not associated with baseline or changes in serum concentrations of IGF-1 and DHEA-S. Beyond its role on somatic growth and development, IGF-1 has a neurotrophic role and is involved in the regulation of synaptic plasticity (Nieto-Estevez et al., 2016). While the liver is the primary site of IGF-1 production and systemic IGF-1 readily permeates the blood-brain barrier, there is also small local production in brain regions, including the hippocampus (Nieto-Estevez et al., 2016). Earlier studies on the relationship between serum IGF-1 and cognition in older adults have yielded mixed evidence, including the interesting observation of a possible U-shaped relationship where both high and low serum concentrations of IGF-1 were associated with poorer cognitive function (Frater et al., 2018). Most of the earlier studies involved healthy older adults, although in a small cohort of older persons with MCI, higher serum IGF-1 was associated with better cognitive performance, while lower IGF-1 concentration was also found to be associated with MCI (Calvo et al., 2013; Doi et al., 2015). However, with the earlier analyses being cross-sectional, it remains uncertain whether individual changes may potentially affect the rate of cognitive decline over time. Our results suggest that disease progression following established clinical manifestation of AD is independent of circulating IGF-1 changes.

Despite biologic evidence for the neuroprotective effects of androgens against amyloid-β induced apoptosis and tau hyperphosphorylation, we found no association between DHEA-S and disease progression. Our findings are consistent with an earlier study in which plasma DHEA-S was not associated with presence of AD, impairment in cognitive domains, or cumulative mortality (Bo et al., 2006), albeit in contradiction to a subsequent study reporting lower plasma DHEA in AD patients respective to age-matched controls (Aldred and Mecocci, 2010). Further, DHEA levels within cerebrospinal fluid were significantly higher in AD patients compared with cognitively normal controls and correlated with Braak neuropathological disease stage. Postulated reasons for the observed elevation include compensatory mechanisms in AD, heightened stress in more severely ill patients with AD, as well as induction by amyloid-β, indicating an adaptive response (Naylor et al., 2008). However, while CSF DHEA levels were similarly elevated in AD relative to controls in a separate study, CSF DHEA-S levels were notably significantly lower, suggesting that DHEA elevation may be consequent to its accumulation from reduced downstream transformation, rather than being a neuroprotective mechanism (Kim et al., 2003). On the contrary, DHEA positively modulates excitatory *N*-methyl-D-aspartate receptors while it negatively modulates inhibitory γ-aminobutyric receptors, potentially driving the excitotoxicity in AD and thus representing a non-adaptive response that may be driving AD pathophysiology (Naylor et al., 2008).

The strength of this study lies in our careful phenotyping and exclusion of participants with other central nervous conditions, such that the findings may be more specific to AD pathology, particularly for MCI which is etiologically heterogeneous. With regards to study limitations, we acknowledge that the relatively small sample size of only 15 progressors in our exploratory study may have contributed to the failure to demonstrate an association between disease progression and inflammatory as well as endocrine biomarkers. However, even with the whole cohort pooled, there was no correlation between serial change in inflammatory-endocrine biomarkers and change in cognitive performance or global disease severity. It also remains to be ascertained whether serum levels of biomarkers may reflect their concentrations in the brain. For instance, while circulating IGF-1 readily crosses the blood-brain barrier, local paracrine production has been postulated to be the major source of IGF-1 within the brain. The possibility of circulating biomarker levels being downstream of ongoing pathological changes in the brain cannot be definitively excluded, particularly with the absence of a normal control group. Further, the lack of cognitively healthy elderly precludes inference on the diagnostic or predictive utility of the examined biomarkers and the pathophysiology driving the initial symptomatic manifestations of AD.

In conclusion, our study provides preliminary clinical evidence for progressively dysfunctional Wnt signaling through DKK-1 antagonism in contributing to disease progression amongst cognitively impaired older adults with MCI and mild-moderate AD. The findings offer a platform to encourage further search for Dkk-1 antagonist molecules as potential therapeutic agents to ameliorate AD progression.

## Data availability statement

The datasets for this manuscript are not publicly available because there is no standing database created for this study. Requests to access the datasets should be directed to laura.tay.b.g@singhealth.com.sg.

## Ethics statement

Informed written consent was obtained from the patient or legally acceptable representative where appropriate, and the study was approved by the Domain Specific Review Board (DSRB) of the National Healthcare Group (NHG). All subjects provided written informed consent, with the consent form having been approved by the institutional review board.

## Author Contributions

LT, MC, and WL contributed to the conception and design of the study. BL performed the blood biomarker analysis. AY contributed to data collection. LT wrote the first draft of the manuscript. BL and WL wrote sections of the manuscript. All authors contributed to manuscript revision, and read and approved the submitted version.

## Conflict of Interest

The authors declare that the research was conducted in the absence of any commercial or financial relationships that could be construed as a potential conflict of interest.
